# Early dye laser treatment of hypertrophic sternotomy scars with prominent vascular component

**DOI:** 10.1016/j.jpra.2026.02.021

**Published:** 2026-02-27

**Authors:** Giovanni Cannarozzo, Laura Pieri, Lara Ronconi, Irene Fusco, Tiziano Zingoni

**Affiliations:** a Lasers in Dermatology Unit, University of Rome Tor Vergata, Via Columbia 2, 00133 Rome, Italy; b Department of Clinical Research and Practice, El.En. Group, Via Baldanzese 17, 50041 Calenzano, Italy

**Keywords:** Dye laser, Hypertrophic sternotomy scars with vascular component, Cardiac surgery, Suture removal

## Abstract

**Purpose:**

A considerable percentage of patients develop hypertrophic scarring due to the healing process after heart surgery. The purpose of this study was to determine whether PDL applied on the day of suture removal, is even more successful in minimizing symptoms that impair the patient’s quality of life and the cosmetic appearance of scars from median sternotomy cardiac surgery procedure. Materials/methods: A total of 12 patients with hypertrophic sternotomy scars resulting from cardiac surgeries underwent 4–5 treatment sessions with Dye laser as soon as the wound’s sutures were taken out. After 40 days, the patient was re-assessed using an in-vivo multispectral skin imaging optical evaluation of the skin structures. Images were captured before and after each treatment, as well as 6 and 12 months after the last treatment. Twelve months after the last session each patient’s level of improvement was assessed using the Vancouver Scar Scale (VSS). Twelve months following the conclusion of treatments, the patients were given photographs and filled out a satisfaction questionnaire.

**Results:**

Analysis of the VSS subscales after 1 year from the last laser treatment showed significant improvement in all parameters examined. Nine patients (75%) were very satisfied, 3 patients (25%) were satisfied, and no patients expressed dissatisfaction or insufficient satisfaction. Relevant side effects were absent. Before and after all treatments, multispectral and hemoglobin analyses showed overall patient’s improvement.

**Conclusions:**

PDL therapy has been proven to physically improve hypertrophic sternotomy scars with few side effects, reducing or even completely avoiding the need for reconstructive surgery.

## Introduction

Wound healing after heart surgery is important for blood loss prevention, skin restoration, and infection management since poor wound healing often leads to problems that are associated with poor survival.^1^ A considerable percentage of patients develop hypertrophic scarring or keloid as a result of this healing process, with a mean frequency of 35% after a year.^2^ Despite the lack of contagious repercussions associated with these scars, they result in reduced quality of life, impaired functionality, and cosmetic issues.^3^ While the exact mechanisms behind abnormal wound healing are still being investigated, inflammation is a major factor in the formation of hypertrophic scars.^4^ Heart surgery is intrinsically associated with a high systemic inflammatory state, and multiple studies have evaluated the effects of different drugs that help manage the inflammatory state that follows surgery.^5^

There have been reports of hypertrophic scarring in 32–72% of cases.^6^

Despite our improving understanding of wound healing, keloids and hypertrophic scars are difficult to avoid and manage. Scars from cardiac surgery, in particular, can have an impact on a patient’s daily life by causing pressure, itching, pain, restricting movement, posing postural difficulties, and occasionally reopening the wound even when the chest moves normally during the process of breathing; additionally, scarring-induced aesthetic issues have been shown to have negative psychological effects, lowering patients’ quality of life and overall productivity. In order to minimize uncomfortable circumstances and the appearance of these scars, both patients and physicians are looking for solutions, and laser treatment could offer an option to those already available.

Treatments for hypertrophic scars and keloids include radiotherapy, cryosurgery, excisional surgery/grafting, silicone gel sheeting, pressure dressings, topical retinoids, intralesional steroids, and 5-fluorouracil (5-FU) injections; however, reports regarding their efficacy have been inconsistent.^7^ Unfortunately, as a result of significant side-effects of various treatment modalities, dyspigmentation, atrophy and recurrence rate, their benefits are limited.^8^

Since they were originally employed for hypertrophic scars and keloids by Apfelberg et al.^9^ and Castro et al.^10^ in the mid-1980s, a growing number of lasers with different wavelengths have been studied. It has been demonstrated that lasers like Nd:YAG laser^11^ the CO2 laser^12^ and pulsed dye laser (PDL)^13^ can effectively improve the aesthetic appearance of hypertrophic scars and keloids.

It is well recognized that pulsed dye laser treatment is an effective way to cure hypertrophic scars with vascular component. This treatment not only lessens the texture and pliability of scars but also their symptoms, such as redness, itching, and pain.^14^

PDL produces visible light pulses with wavelengths of 585 or 595 nm. Oxyhemoglobin is the primary absorber of these light pulses, resulting in selective blood vessel destruction while sparing the skin above. PDL is not only the gold standard treatment for vascular lesions such as superficial hemangiomas, port-wine stains, and telangiectasias, but it has also been demonstrated to be effective in treating nonvascular lesions that rely on blood flow. Indeed, PDL therapy targets the vascular component of scars, reducing erythema and improving overall color.^15^ The majority of hypotheses include selective photothermolysis, in which hemoglobin absorbs light energy from vascular lasers (like PDL), causing heat and coagulation necrosis. Hemoglobin absorbs light from the PDL, which has an emission wavelength of 585 to 595 nm.^16^ This causes hypoperfusion and hypoxia, which can lead to the release of histamine or other chemicals that affect fibroblast activity, as well as neocollagenesis.

Despite the fact that PDL has been used for the past 20 years to treat hypertrophic scars with generally positive outcomes, we present clinical cases where scars were improved due to the prompt use of Dye laser application; in fact, it is used early in cardiac surgery scar’s maturation, i.e., within 30 days after suture removal. The purpose of this study was to determine whether PDL applied on the day of suture removal is even more successful in minimizing symptoms that impair the patient’s quality of life and the cosmetic appearance of scars from the median sternotomy cardiac surgery procedure.

## Materials and methods

### Study design and patient selection

From January 2021 to January 2023, 12 patients (8 females, 4 males; mean age 43.9 (±6.3) years old) with hypertrophic sternotomy scars resulting from cardiac surgeries, with skin types I–IV and with no contraindications to laser treatment (pregnancy, photosensitivity, history of skin cancers), were enrolled at University of Tor Vergata in Rome (Italy) in the Lasers in Dermatology Unit.

All patients met the same predefined clinical eligibility criteria before laser initiation, ensuring comparable baseline conditions across the study cohort.

Patients with clinical signs of surgical site infection, wound dehiscence, or delayed healing were excluded from the study. All patients received treatment following a thorough clinical anamnesis (skin type, clinical manifestations, previous history or family history of hypertrophic scars and keloids, health conditions, prior medications, and lifestyle); specifically, there was no family history or prior history of hypertrophic scars and keloids among the patients. Data was retrospectively collected and analyzed. The patients received also a signed informed consent form outlining the procedure’s risks and for the use of their clinical data and photos.

### Dye laser treatment protocol and post-treatment care

Upon wound’s sutures removal, the patient started receiving laser treatment. All surgical laser procedures were performed using a standardized technique, and without the use of tension-relieving devices or adjunctive interventions that could influence scar outcomes.

Before receiving treatment, the affected area had been cleaned to remove any contaminants (lotions, ointments, etc.) that might have reacted with light radiation. To determine the appropriate therapy for each patient, a skin test was conducted prior to the start of treatment.

The skin was treated with a topical anesthetic cream (Ropivacaine 2 mg/mL solution, Naropin; Aspen Pharma Trading Limited) for 30 min prior to the laser treatment and the skin cooling system (Cryo6, Zimmer) was used before, during and after the procedure. Patients didn’t receive any additional treatments (e.g., topical medications, pressure dressings, or systemic anti-inflammatory agents) during or after laser sessions.

For hypertrophic scars with a vascular component, the treatment was initiated with dye laser therapy (Synchro VasQ, DEKA M.E.L.A., Calenzano, Italy) using fluence 6.5–11 J/cm2, pulse duration 0.5 ms and spot size of 10–12 mm.

In order to avoid potential cutaneous infections and crusting, an antibiotic ointment containing fusidic acid was also applied to the target areas for 7 days following each laser session; 4–5 treatment sessions were given to each patient.

On every session, complications and side effects were also noted; potential adverse events include immediate post-laser purpura, recurrence and infection. Patients were instructed not to use hot water on the treated area, play sports or be in direct sunlight for at least 48 h.

Images were captured using a Canon digital camera and a polarized flash (Anthology system, DEKA M.E.L.A., Calenzano, Italy) before and after each treatment, as well as 6 and 12 months after the last treatment; photographs were standardized using identical cameras, shooting settings, same flash and ambient light. The patient was also evaluated using an in-vivo multispectral skin imaging optical evaluation (Antera 3D; Miravex Limited, Dublin, Ireland) of the skin structures to track the effects of treatment analysis. Antera 3D evaluates the melanin and vascular components by reconstructing skin surfaces with the aid of a computer and multidirectional lighting.

Twelve months after the last session each patient’s level of improvement was assessed using the Vancouver Scar Scale (VSS). The VSS evaluates scar’s vascularity, height, pliability and pigmentation; numbers were assigned to each of the four parameters, previously listed. To get the final VSS score, the scores from each parameter are added together.

Twelve months following the conclusion of treatments, the patients were given photographs and asked to rate their subjective impression of the overall outcome on a satisfaction questionnaire (dissatisfied, not very satisfied, satisfied and very satisfied).

Data were summarized using mean and standard deviation and Student’s T-test was used to test clinical data. The level of significance for statistical tests was 0.01.

## Results

The average VSS score assessed by the physician significantly (*P* < 0.001) decreased from 8.9 ± 1.9 at baseline to 3.4 ± 1.8 at 1 year from the last laser treatment. Analysis of the VSS subscales (erythema, pliability, height, and pigmentation) after 1 year from the last laser treatment showed significant improvement in all parameters examined.

Global improvement was noted by all patients, as shown by a significant decrease in scar erythema, height, rigidity and scar redness (deep red or purple color). Patients were asked to rate their subjective satisfaction with the outcome: 9 patients (75%) were very satisfied, 3 patients (25%) were satisfied, and no patients expressed dissatisfaction or insufficient satisfaction.

Relevant side effects such as crusts, blisters, atrophy, and scars were absent in every case; purpura, the most frequent adverse side effect, manifested in every patient and resolved in 7–10 days. Surface cooling markedly diminished these side effects.^17^^,^^18^ Swelling and erythema are frequently present immediately after treatment, but resolve within 24–48 h. No cases of transient hypopigmentation and vesicle formation were reported.

**Figure 1:**
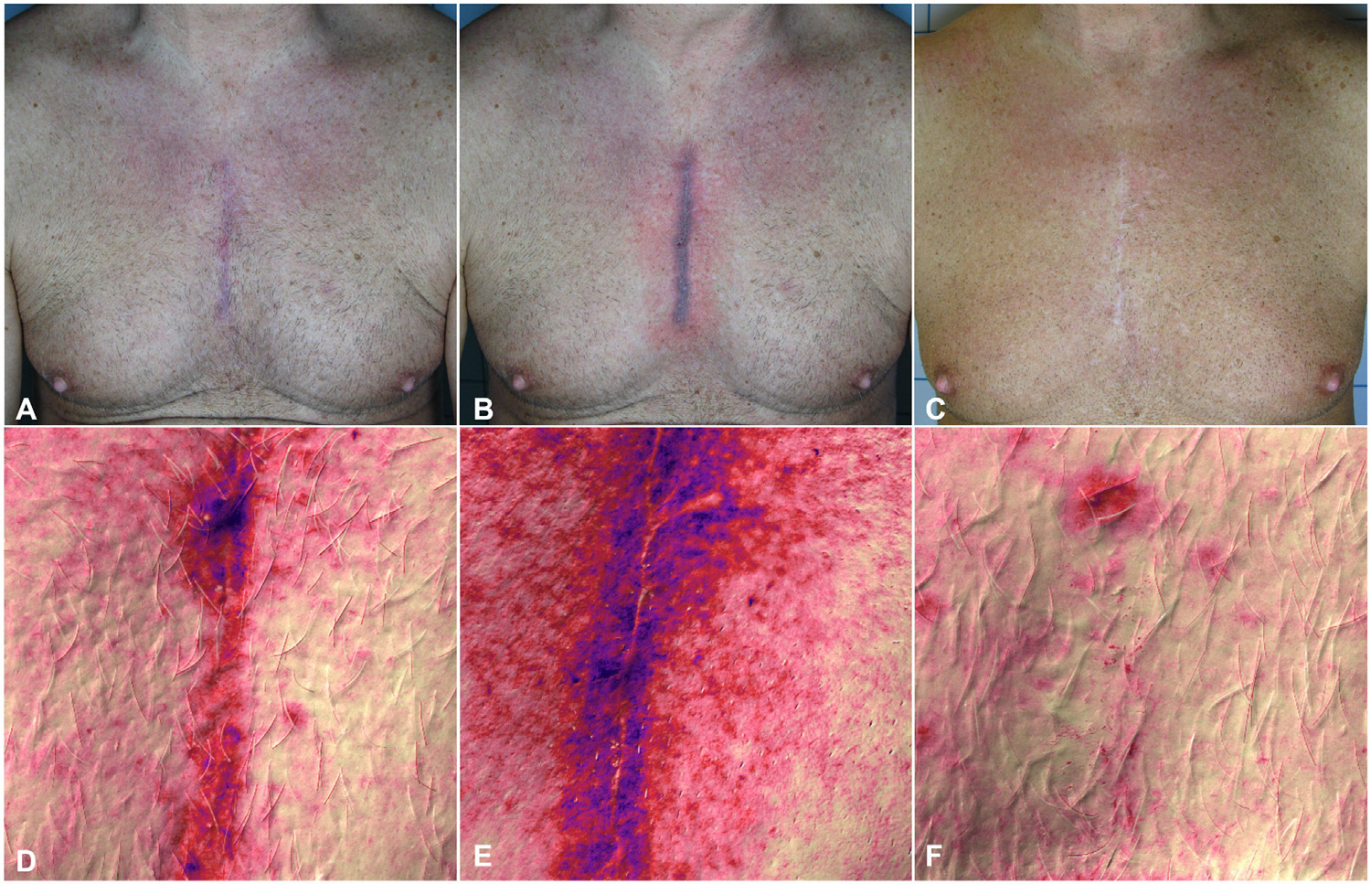
Clinical and multispectral skin imaging optical photographic evaluation of Hypertrophic Sternotomy Scars of male patient derived from cardiac surgery procedure. Images were acquired before (A, D) immediately after the laser treatment session (B, E) and at 1 years follow up from the last laser treatment session (C, F). Red in multispectral pictures produced with Antera 3D (D, E, F) denotes vascular activity and scar inflammation, whereas blue denotes pigment changes.

**Figure 2:**
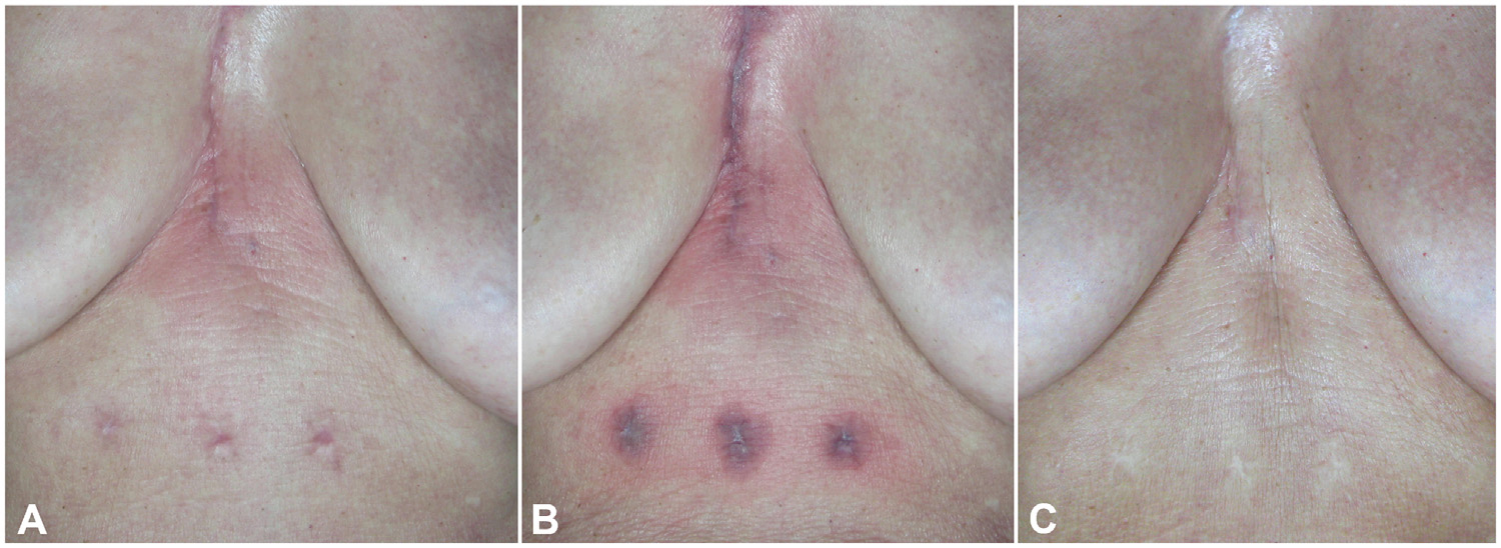
Clinical skin imaging of Hypertrophic Sternotomy Scars of female patient derived from cardiac surgery procedure. Clinical images were acquired before (A) immediately after the laser treatment session (B) and at 1 years follow up from the last laser treatment session (C).

**Figure 3:**
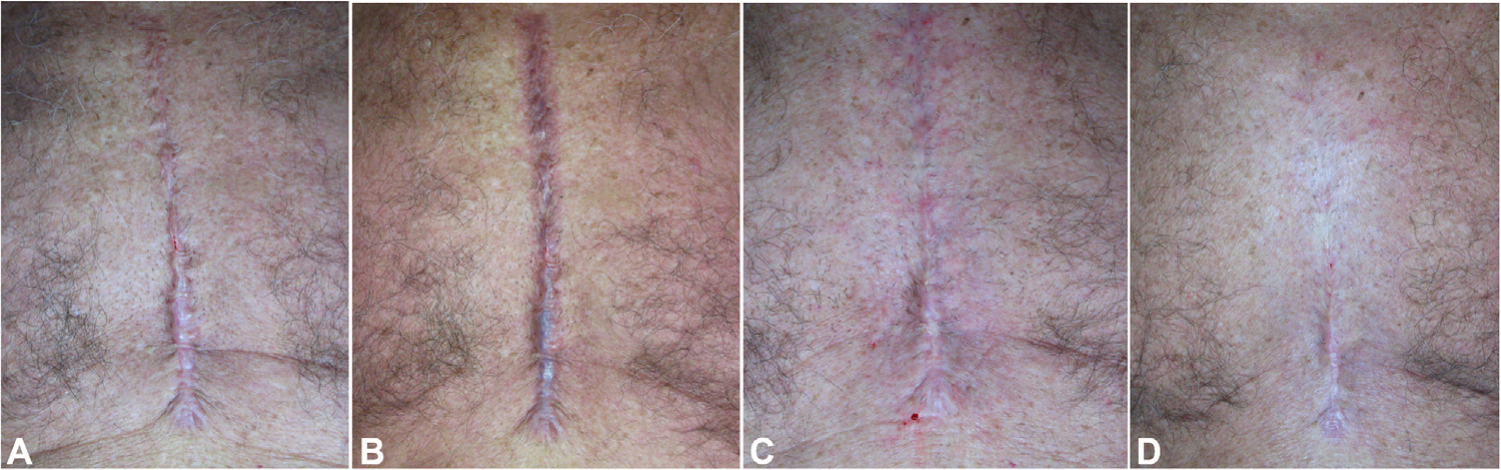
Clinical skin imaging of Hypertrophic Sternotomy Scars of male patient derived from cardiac surgery procedure. Clinical images were acquired before (A) immediately after the laser treatment session (B), at 6 months follow up (C) and at 1 years follow up from the last laser treatment session (D).

Figures 1, Figure 2, Figure 3 show cases of successful treatment of cardiac surgery scars; multispectral pictures created with Antera 3D were collected before and after each treatment, and all patients showed improvement in pigment changes and overall scar improvement, including decreased vascular activity and inflammation (Figure 1D, E and F).

## Discussion

The basic biochemical aspects of keloid and hypertrophic scar development have been suggested to be an imbalance between matrix breakdown and collagen biosynthesis, resulting in an excessive buildup of collagen in the wound. Nevertheless, the specific source of these skin lesions is unknown. Fibroblasts generate new extracellular matrix components, initiate collagen synthesis, and employ desmin, actin, and other contractile proteins to create tension near the wound border. Compared to normal fibroblasts, keloid and hypertrophic scar-derived fibroblasts produce more collagen per cell.^19^ Therefore, suppressing the excessive and uncontrollable fibroblast activity in hypertrophic scars and keloids may be crucial to treating this aberrant wound response.

Although the use of intralesional formulas resulted in a quicker response, the risk of side effects from intralesional corticosteroid therapy is significantly higher.^20^

It has been demonstrated that intralesional injection of 5-FU has favorable effects on scar treatment, including a decrease in scar volume and improvement in symptoms. But within a year of treatment, there are recognized side effects and recurrences.^21^

There is disagreement among researchers on the effectiveness of monotherapy in treating hypertrophic scars and keloids; it is frequently discovered that numerous treatments are far more successful.^22^ Indeed, when corticosteroid therapy was combined with 5-fluorouracil (5-FU), these agents were more effective and safer.^23^ Recurrence is often evaluated inconsistently and inadequately in combination therapy studies, sometimes with shortened follow-up duration. Long-term monitoring (18–24 months) is necessary to define recurrence in the context of treating pathologic scars with various intralesional agents, even though scar recurrence can happen up to a year following therapy.^24^

All research participants with hypertrophic heart surgery scars showed substantial improvement in both clinical and histological parameters after receiving treatment with 585-nm PDL irradiation.^25^

Three distinct stages constitute the wound healing process after surgery: The proliferative process, the inflammatory reaction, and tissue remodeling.^26^

The most recent research consistently reveals new aspects of the complex interactions that occur during wound healing between integrins, inflammatory cytokines, and angiogenic agents.^27^^,^^28^ These interactions include a wide range of self-regulating interactions between different cell types, such as macrophages, neutrophils, fibroblasts, mast cells and vascular endothelial cells. Heat shock protein (Hsp-47 and Hsp27), TGF-beta, vascular endothelial growth factor (VEGF), connective tissue growth factor (CTGF), platelet-derived growth factor (PDGF), and insulin-like growth factors (IGF) have all been found to be crucial for the synthesis of collagen and fibroblast proliferation. These findings have led to an investigation into the effects of various growth factors on the development of keloid formation.^29^ Specifically, TGF-beta expression is limited to pathological fibrotic tissue, such keloids or hypertrophic scars, and is not present in normal tissue; CTFG directly increases the fibroblasts’ proliferation and extracellular matrix (ECM) formation.^30^

PDL is a painless, non-invasive procedure that does not require anesthesia. PDL uses selective photothermolysis to target blood vessels with minimum collateral damage; the short pulse duration limits the amount of local thermal injury caused by hemoglobin’s preferential absorption of PDL energy, resulting in thrombosis, vasculitis, and progressive healing. The exact mechanism by which PDL impacts scarring is unknown, although it is thought that ischemia produced by microvascular damage alters collagen or collagenase release, or that it can deplete a scar of nutrients, preventing scar growth. It is well recognized that hypertrophic scars and keloids can be successfully treated using pulsed dye laser treatment, which not only lowers scars’ texture and pliability but also their symptoms including itching, redness and pain.^31^ Alternatively, sufficient heat may be sent straight from the blood vessels to the surrounding dermis, altering the composition of the scar’s collagen; when heat breaks the disulfide bonds connecting its fibers, collagen can realign itself. Heat causes the release of histamine and other biochemical components, which may also affect fibroblast activity.^23^ This biochemical process is linked to the laser’s efficacy. Kuo et al.’s biochemical studies show that PDL treatment of keloid tissue results in decreased TGF beta-1 induction, increased matrix metalloproteinase (MMP) expression, and increased interleukin-6 (IL-6).^24^ PDL also raises the activity of p38 mitogen-activated protein kinase and extracellular signal-regulated kinase, and it promotes collagen breakdown and fibroblast death. Additionally, it has been shown that PDL reduces collagen type III deposition and fibroblast proliferation.^14^^,^^32^ The parameters of the laser protocol adopted in this study were determined on the basis of previously published clinical evidence^32^ and established clinical practice.

In these cardiac surgery scars, higher fluences are used to target deeper structures due to the optimization of laser devices, parameters, and settings. According to Bruscino et al., dermatologists can now get quicker outcomes by utilizing cooling systems and longer pulse widths and wavelengths.^33^ Choosing the appropriate PDL fluence is essential since low-fluence PDL can also accelerate the production of procollagen, while high-fluence PDL can promote focal dermal coagulation and increase the risk of side effects, especially in those with darker skin.^34^ However, some writers have found that a better tissue response is linked to a higher PDL fluence and smaller spot size.^35^

Early intervention, in our opinion, is critical when treating heart surgery scars, and it would be even ideal to begin PDL treatment immediately after the sutures are removed. According to our observations, all of the patients who did not respond had a longer history of scarring. Indeed, the efficacy of PDL is restricted by the thickness of the pre-existing lesion, where the vascular component is too inadequate or non-existent. Although PDL only penetrates to a depth of around 1.2 mm, hypertrophic scars or keloids thicker than 1 cm may only show marginal improvement. In these circumstances, combination therapy, which uses a dye laser in addition to CO2 micro-ablation, is the most successful course of treatment. The CO2 laser uses controlled in-depth micro-ablations on the scar to debride the vascular component and visually prepare it for the subsequent dye laser treatment. The PDL treatment, when associated with the CO2 laser, modifies the inflammatory and regenerative processes to promote non-hypertrophic healing and lowers the vascular component.^36^^,^^37^ Both lasers need to be used in the same session, even though the PDL regulates the healing process and reduces fibrotic deposition most efficiently when the inflammatory process is still going on.^38^

An interesting observation was made in every patient receiving PDL treatment for cardiac surgery scars on the same day as suture removal: A major cosmetic component of the observed improvement is an amelioration in scar skin texture; these changes in texture are thought to be the result of collagen remodeling. These improvements enhance the patient’s appearance as well as their overall quality of life. Specifically, the better scar decreases pressure, pain, itchiness, movement restriction, postural problems, and wound reopening during inhaling. Similar to our investigation in the study of Nouri and colleagues, scars were treated with PDL immediately after suture removal demonstrating that this procedure is a safe and efficient way to enhance the aesthetic appearance and quality of surgical scars.^13^

Clinically, early hypertrophic scars and keloids may present with overlapping features, particularly in the initial stages of scar development, making differentiation challenging. Hypertrophic scars are typically confined to the original wound margins and may show partial regression over time, whereas keloids tend to extend beyond the wound boundaries and demonstrate persistent or progressive growth. In the present context, the distinction was therefore based primarily on clinical characteristics and growth patterns rather than definitive histopathological criteria. The lack of long-term follow-up and histological analysis represents a limitation, as these factors could have provided more conclusive evidence for accurate classification.

The small sample size also represents a limitation of this study; however all of the patients’ aesthetic results significantly improved, indicating the potential of this laser technique. Furthermore, following treatment sessions, none of the patients we saw noticed any discomfort or long-term side effects.

In order to optimize clinical benefit, we suggest early intervention following sutures removal, based on this data.

Additionally, the absence of a control group and the limitation of the follow-up to 12 months undoubtedly represent further limitations; furthermore, a future experiment with a control group, additional patients, standardized and quantitative baseline wound assessments, and possibly long-term surveillance (18–24 months) will be planned. Finally, PDL therapy with higher fluences is especially effective thanks to its remarkable aesthetic outcomes; indeed, not only does it make hypertrophic sternotomy scars physically better with few side effects, but it may also reduce or even completely avoid the need for reconstructive surgery. Repigmenting the light white line that frequently remains in the scar will be our aim in future research.

The CO2 family lasers, which were developed to achieve deeper ablation depths, can be used with the new scanner because laser emission penetrates the dermis very deeply, as demonstrated by pre-clinical studies using ex-vivo animal models (sheep skin), ex-vivo human skin, and clinical studies on acne scars.^39^^,^^40^ Since many scar regions are deeper than a few millimeters, a laser ablation penetration depth is required to initiate the healing cascade and provide successful scar remodeling outcomes.^39^ Consequently, for future investigation, we plan to employ a new scanner in the ultimate management of cardiothoracic scars.

## Conclusion

PDL therapy has shown to physically improve hypertrophic sternotomy scars with few side effects, reducing or even completely avoiding the need for reconstructive surgery. Larger samples with longer follow-up, such as randomized studies with a control group, are needed to confirm the benefits of early intervention of laser technology in surgical scars.

## Author contributions

Conceptualization, GC, TZ; methodology GC, TZ; software, GC, LR, TZ; validation, GC, LP, LR, IF, TZ; formal analysis, GC,TZ; investigation, GC, LP, LR, TZ; resources, GC, LP, LR, TZ; data curation, GC, LP, LR, IF, TZ; writing—original draft preparation, GC LP, LR, IF; writing—review and editing, GC, LP, LR, IF, TZ; visualization, GC, LP, LR, IF, TZ; supervision GC, LP, LR, IF, TZ,; project administration, GC, TZ; funding acquisition, GC, TZ. All authors have read and agreed to the published version of the manuscript.

## Funding

This research received no external funding.

## Informed consent statement

Informed consent was obtained from all subjects involved in the study.

## Data availability statement

Data that support the study findings are available on request from the corresponding author (IF).

## Declaration of Competing Interest

Authors LP, LR, TZ and IF were employed by El.En. Group. The remaining authors declare that the research was conducted in the absence of any commercial or financial relationships that could be construed as a potential conflict of interest.
